# Too Little Sleep

**Published:** 1872-10

**Authors:** 


### TOO LITTLE SLEEP.

Students, as a class, do not sleep enough. There is no law so fundamental and imperative on the student as the law which requires him to sleep, and no other law does he so systematically and recklessly ignore.

It is a popularly accepted fallacy that students and literary men do not require as much sleep as mechanics and laborers. Physiology shows us that, during the operation of the intellect, rapid changes of tissue take place, and that a few hours of close application to thought and study exhaust the system more than two or three times the same period devoted to manual labor. It is evident, then, in order to compensate for this greater waste of tissue, that the brain worker will require more sleep than the muscle worker.

In the violation of this first great hygienic commandment is found the secret of most of the special diseases to which the student is liable. To this cause can be traced the eye affections that are so common. By neglecting to obtain sufficient rest, the system becomes relaxed and its tone lowered, thereby inviting disease, of which these organs, being especially overtasked and weakened, are the first to become sensible.

